# Autophagy-amplifying nanoparticles evoke immunogenic cell death combined with anti-PD-1/PD-L1 for residual tumors immunotherapy after RFA

**DOI:** 10.1186/s12951-023-02067-y

**Published:** 2023-10-03

**Authors:** Shushan Zhang, Yongquan Huang, Songying Pi, Hui Chen, Feile Ye, Chaoqun Wu, Liujun Li, Qing Ye, Yuhong Lin, Zhongzhen Su

**Affiliations:** Department of Ultrasound, The Fifth Affiliated Hospital of Sun Yat-Sen University, Meihua East Road, No. 52, Zhuhai, 519000 Guangdong Province China

**Keywords:** Incomplete radiofrequency ablation (IRFA) for hepatocellular carcinoma, Autophagy, Zeolitic imidazolate framework-8 (ZIF-8), Immunogenic cell death (ICD), Anti-PD-1/PDL1 therapy

## Abstract

Incomplete radiofrequency ablation (IRFA) triggers mild protective autophagy in residual tumor cells and results in an immunosuppressive microenvironment. This accelerates the recurrence of residual tumors and causes resistance to anti-PD-1/PDL1 therapy, which bringing a great clinical challenge in residual tumors immunotherapy. Mild autophagy activation can promote cancer cell survival while further amplification of autophagy contributes to immunogenic cell death (ICD). To this regard, we constructed active targeting zeolitic imidazolate framework-8 (ZIF-8) nanoparticles (NPs) loaded with STF62247 or both STF62247 and BMS202, namely STF62247@ZIF-8/PEG-FA (SZP) or STF62247-BMS202@ZIF-8/PEG-FA (SBZP) NPs. We found that SZP NPs inhibited proliferation and stimulated apoptosis of residual tumor cells exposed to sublethal heat stress in an autophagy-dependent manner. Further results discovered that SZP NPs could amplify autophagy in residual tumor cells and evoke their ICD, which dramatically boosted the maturation of dendritic cells (DCs). Through vaccination experiments, we found for the first time that vaccination with heat + SZP treatment could efficiently suppress the growth of new tumors and establish long-term immunological memory. Furthermore, SBZP NPs could remarkably promote the ICD of residual tumor cells, obviously activate the anti-tumor immune microenvironment, and significantly inhibit the growth of residual tumors. Thus, amplified autophagy coupled with anti-PD-1/PDL1 therapy is potentially a novel strategy for treating residual tumors after IRFA.

**Graphical Abstract:**

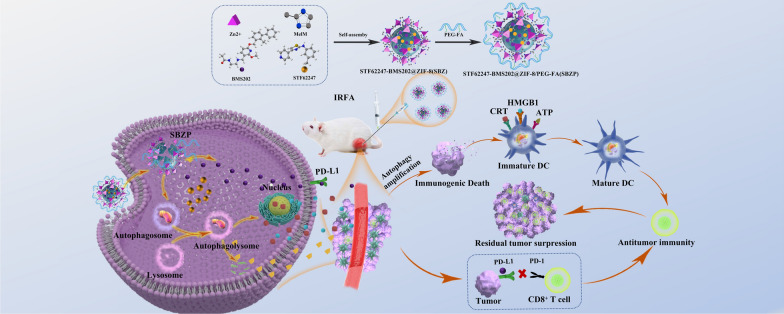

**Supplementary Information:**

The online version contains supplementary material available at 10.1186/s12951-023-02067-y.

## Introduction

Liver cancer is the sixth most common cancer and the third leading cause of cancer-related death worldwide [1, 2]. Hepatocellular carcinoma (HCC) is the most typical primary malignant liver cancer. Radiofrequency ablation (RFA) is one of the main curative therapies in treating HCC for its safety, shorter hospital stay, and fewer complications [3, 4]. However, factors such as complex spatial structure, indistinct border of tumors, and failure to access the ablative margin may lead to incomplete radiofrequency ablation (IRFA), resulting in residual tumors and even accelerating their progression [5, 6]. Research has shown that patients demonstrate an overall recurrence rate as high as 63.5% five years after RFA, with a poorer long-term prognosis than those undergoing surgical resection [7]. How to effectively suppress residual tumors is a major clinical challenge.

RFA releases the tumor debris whilst the tumor burden is mitigated, which stimulates the host’s anti-tumor immune response [8–10]. However, the immunogenicity elicited by RFA is too weak to activate sustained antitumor immune response and suppress the recurrence of residual tumors [9]. In recent years, RFA combined with immunotherapy has been employed to treat HCC in various clinical studies, demonstrating significant clinical value [11]. Immune checkpoint blockade (ICB) is one of the most promising approaches to activating antitumor immunity. It recovers the once-suppressed ability of T cells to recognize tumors by blocking PD-1 from binding with PD-L1, which then activates systemic immunity [12]. Nevertheless, the latest research has revealed that increased tumor-infiltrating myeloid cells after IRFA strengthened the immunosuppression microenvironment of residual tumors [13, 14], which severely reduced the sensitivity of residual tumors to ICB therapy [15–17].

Autophagy plays a paradoxical role in anti-tumor immune response [18, 19]. Low-level autophagy is cytoprotective and conducive to the survival of tumor cells with restriction on the release of immunogenicity. It leads to the formation of an immunosuppressive microenvironment, favoring the survival of regulatory T cells (Tregs) and the polarization of tumor-associated macrophages (TAMs) [20, 21]. Contrastively, high-level autophagy is able to promote cell death in tumor cells and release damage-associated molecular patterns (DAMP) from dead cells, which can remodel the tumor microenvironment (TME) and activate anti-tumor immunity. Studies showed [22–25] that sublethally heated HCC cells exhibit a low level of autophagy activation after IRFA, serving as self-protection that would induce immune tolerance with resistance to immunotherapy. Multiple preclinical research has confirmed that sufficient intracellular autophagy activation can induce autophagy-dependent cell death (ACD) [26], also known as Type II programmed cell death. It was also reported that enhanced activation of autophagy can release immunogenicity to strengthen anti-tumor immunity while inducing ACD [27]. Moreover, sublethally heated residual tumors are pathologically hyper-sensitive to autophagy compared to normal cells [28]. Therefore, in contrast to inhibiting protective autophagy, amplified activation of autophagy can not only eliminate low-level autophagy’s protection on HCC cells after IRFA [29], but also evoke immunogenic cell death (ICD) in residual tumor cells and reverse the immunosuppressive microenvironment [30, 31]. Amplified activation of autophagy combined with ICB may have synergistic effects and improve efficacy against residual tumors after IRFA.

We previously reported that the extensive angiogenesis after IRFA led to an augmented permeability and retention (EPR) effect and contributed to the enrichment of nanodrug in residual tumors [32]. ZIF-8 is a metal–organic framework synthesized by Zn^2+^ and 2-MeIM with good biocompatibility and perfect acid-induced degradability. It has the advantages of variable pores, easily-modified structure, and high drug-loading capacity [33–35]. Aiming to tackle the clinical challenge of insufficient immunogenicity and decreased microenvironmental sensitivity to immunotherapy after IRFA [14], autophagy inducer STF-62247 (STF) and small molecule inhibitor of the PD-1/PD-L1 interaction BMS202 (BMS) were loaded into the ZIF-8 framework via one-pot synthesis to prepare STF62247-BMS202@ZIF-8/PEG-FA (SBZP) NPs. STF is a small molecule that induces autophagy, which could powerfully transformed protective autophagy into ICD and remodeled the immune microenvironment [36–38]; BMS is a small molecule inhibitor of the PD-1/PD-L1 interaction which could bolstered the ability of T cells to fight against tumors [39, 40]. Overall, we report a novel strategy of amplified activation of autophagy in combination with ICB for residual cancer treatment after IRFA, which may have immense potential in the future.

## Material and methods

### Chemical reagents

Zn (NO3)2•6H2O and 2-MeIM were purchased from Aladdin Holdings Group Co., Ltd (Shanghai); PEG-FA 2000, ICG, and FITC from Rixi Biotechnology Co., Ltd. (Xi’an); BMS202 (HY-19745), STF62247 (HY-100746), 3-MA (HY-19312), and Chloroquine (HY-17589A) from MedChemExpress (Shanghai); ethanol from Guangzhou Chemical Reagent Factory.

LC3B antibody (L7543) was purchased from Sigma; ATG5 antibody (T55766) from Abmart; p-mTOR antibody (Ser172) (#2971S) from CST; HMGB1 antibody (10,829-1-AP) from Proteintech; HRP-Goat anti-rabbit IgG(H + L) (E030120-01) from Earthox; CD4 antibody (bs-0647R), CD8 antibody (bs-4791R) from Bioss;p62 antibody (ab109012), mTOR antibody (ab137133), Calreticulin antibody (ab92516), Ki67 antibody (ab16667), Goat anti-rabbit IgG H&L (Alexa Fluor® 488) (ab150077), Goat anti-rabbit IgG H&L (Alexa Fluor® 555) (ab150078), and DAPI Staining Solution (ab228549) from Abcam.

### The preparation of STF@ZIF-8/PEG-FA (SZP), BMS@ZIF-8/PEG-FA(BZP), and STF-BMS@ZIF-8/PEG-FA (SBZP)

Solution A was prepared by dissolving 300 mg of Zn (NO3)2•6H2O in 8 mL of ethanol, while solution B by dissolving 320 mg of 2-MeIM in 8 mL of ethanol. 4 mL of STF (5 mg/mL) and/or 4 mL BMS (1 mg/mL) were added dropwise into solution A respectively (stirred at 1200 rpm for 5 min, room temperature). Then, the above solutions were added dropwise to 8 mL of solution B and stirred at 1200 rpm for 15 min at room temperature. The NPs were purified by centrifugation (10,000 rpm) and washed with ethanol 3 times. After vacuum drying, SZ, BZ, and SBZ came into being. 50 mg of SZ, BZ, and SBZ were added to 5 mL of deionized water containing 50 mg of PEG-FA, respectively, then ultrasonically oscillated until they were evenly distributed, and stirred at 1200 rpm for 24 h. The NPs were purified by centrifugation (10,000 rpm) and washed with ethanol 3 times, and finally SZP, BZP, and SBZP were prepared after vacuum drying. By UV spectrophotometer, STF showed a characteristic absorption peak near 295 nm, BMS labeled with FITC, FITC- BMS showed a characteristic absorption peak near 512 nm. The drug loading content (DLC) and drug loading efficiency (DLE) of SBZP NPs were calculated according to the previously reported formulas [23]. The DLC and DLE of STF in SBZP were 19.75% and 86.93%, while BMS was 11.52% and 86.47%.

### Structural characterization of NPs

The morphology and size of NPs were examined using JEM-2100F transmission electron microscope under an accelerating voltage of 200 kV. X-ray diffractometer (XRD, D-MAX 2200 VPC Diffractometer, Rigaku, Japan) was employed to measure the crystal structure. The zeta potential of the NPs was explored by Zetasizer Nano ZSE (Malvern, UK). UV–Vis spectrometer (LAMBDA 365, PerkinElmer, USA) recorded the ultraviolet–visible spectroscopy of NPs. The functional groups were observed by Fourier-transform infrared spectroscopy (FTIR, Bruker, Billerica, MA, USA). The chemical composition was identified via X-ray Photoelectron Spectroscopy (XPS, ESCALAB 250, Thermo Fisher, US). Thermogravimetric analyzer (Pyris, PerkinElmer) measured the mass as the temperature changed from 25℃ to 700℃. The procedure was performed in nitrogen atmosphere and at a constant heating rate of 10℃/min.

### In-vitro experiment on drug release

5 mg of SBZP were dispersed in 20 mL of PBS (pH 5.5 and pH 7.5). UV-Vis absorbance spectrometer was measured at 295 nm. Standard curve of STF was made based on different concentrations in different pH solutions. Then, the STF concentration of SBZP in the sample solutions was extrapolated from the STF standard curve. To measure the BMS content in SBZP, BMS was labeled with FITC. The same method was applied to measure the BMS concentration of SBZP at 512 nm with a fluorescence spectrophotometer.

### Cell lines and cell culture

HCC cell lines (SMMC7721, Huh7, HepG2, Hepa1-6) were obtained from the Cell Bank (Academica Sinica, Shanghai, China) and H22-Luc cells were purchased from Shanghai Fuheng Biotechnology. SMMC7721, Huh7, HepG2, and Hepa1-6 were cultured in DMEM (Gibco-Invitrogen) and H22-Luc cells in RPMI 1640 (Gibco-Invitrogen). All culture media were supplemented with 10% FBS (Invitrogen) and 1% penicillin/streptomycin (Invitrogen). Cell lines were cultured in a humidified incubator at 37 °C and 5% CO_2_.

### Sublethally heated cell model in vitro

Culture dishes sealed with films were heated at different temperatures (43, 45, 47, and 49 °C) for 15 min and different periods of time (5, 10, 15, 30, and 60 min) at 47 °C [41]. Upon being heated, they were transferred to the incubator for 24 h recovery at 37 °C and 5% CO_2_. Cells cultured at 37 °C were selected as the control group.

### IRFA subcutaneous tumor model

H22-Luc cells (5 × 10 ^5^ per mouse) were subcutaneously into the right thigh of female BALB/c mice (aged 6 weeks, Guangzhou Yancheng Biotechnology, China). When the tumors grew to 200-400mm^3^, the mice were anesthetized and placed on the VIVA grounding pad. RF electrodes were inserted into the tumor and one-third of it was pierced under ultrasound guidance. IRFA was performed with a power output of 5W for 20 s. D-luciferin potassium salt (PerkinElmer, USA) was intraperitoneally injected for bioluminescent imaging in H22 tumor-bearing mice.

### Cell viability and proliferation assays in vitro

HCC cells were seeded in 96-well plates (5,000 cells/well), incubated with various concentrations of drugs (STF and SZP) and different reagents (PBS, ZIF-8, STF, SZP, SZP + 3MA), and subsequently heated following the method described in Section “Sublethally heated cell model in vitro”. After 24 h or 48 h rewarming, the cells were cultured with 10% CCK8 (DOJINDO, Kumamoto, Japan) solutions. A multimode reader (Synergy HTX, Bio-Rad, USA) was used to record the absorbance value at 450 nm, and then, the curve of cell growth was drafted with cell viability and IC50 value calculated.

### Cell apoptosis analysis and double staining using live/dead cell viability assay kit

Sublethally heated cells treated by different drugs were stained with Annexin V-FITC Apoptosis Detection Kit (BD Biosciences, USA) and Calcein-AM/PI Dual Staining Kit (Solarbio, Beijing, China). Apoptosis and cell death rates were measured using Cyto-FLEX LX Flow Cytometer (Beckman Coulter, USA). Collected data were analyzed with FlowJo 7.6.5 (Tree Star, USA). The fluorescent signals of live and dead cells were observed with an inverted fluorescence microscope (CLSM IX73, Olympus, USA).

### Protein immunoblotting

Total protein was extracted using the RIPA Lysis and Extraction Buffer (Thermo Fisher, Massachusetts, USA). 20 μg of protein was separated by SDS-PAGE and transferred onto PVDF membrane (Bio-Rad, CA, USA), which then was sealed with 5% skim milk at room temperature for 1 h. The membrane was incubated with primary antibodies (anti-GAPDH 1:5000, anti-β-actin 1:5000, anti-LC3B 1:1000, anti-P62 1:1000, anti-p-mTOR 1:500, anti-mTOR 1:500, anti-HMGB1 1:1000, and anti-ATG5 1:1000) at 4 °C overnight, and then with hHRP-conjugated secondary antibodies (1:5000) at room temperature for 1 h. Chemiluminescence gel imager (ChemiDoc XRS + , Bio-Rad, USA) was used to detect proteins on the membrane.

### Cellular drug uptake

SMMC7721 cells were seeded in 24-well plates (5 × 10^4^ cells/well) and incubated with FITC@ZIF-8 NPs for 2 h, 4 h, 8 h, 12 h, and 24 h, respectively. Subsequently, the cellular uptake ratio and intracellular distribution of ZIF-8 NPs were evaluated by confocal laser scanning microscopy (CLSM) and flow cytometry.

### The accumulation and distribution of NPs in tumors

ICG@ZIF-8/PEG-FA NPs and free ICG were injected into the H22 tumor-bearing mice via the tail vein, respectively. Fluorescent images were captured at different time points using IVIS imaging system (IVIS Lumina, PerkinElmer, USA). The tumor-bearing mice were euthanized to obtain tumors and important organs for fluorescent imaging after 24 h. At the same time, a few tumor tissues and organs were cauterized and nitrified, and subsequently the concentration of [Zn^2+^] was determined by 5800 ICP-OES (Agilent, Japan).

### Autophagy-related in-vitro experiments

#### Transfection with mRFP-GFP-LC3 plasmid for monitoring autophagic flux

According to the manufacturer’s protocol, HCC cells (SMMC7721, Huh7) were seeded in 6-well plates containing cover slides at density of 2 × 10^5^ cells/well and transfected with 2500 ng of mRFP-GFP-LC3 plasmid (Hanbio, China Shanghai) using Lipofectamine 3000. The GFP and mRFP blot were observed by CLSM (ZEISS LSM880, Germany). Yellow blot represented autophagosomes (merged of mRFP and GFP), while red blot indicated autolysosomes (only mRFP).

#### Transmission electron microscope (TEM)

Sublethally heated cells separately treated with PBS and SZP were collected and fixed in 2.5% glutaraldehyde for 2-4 h. They were rinsed by PBS 3 times, fixed in 1% osmium tetroxide at 4 °C for 2 h, dehydrated with gradient ethanol, transitioned in epoxypropane, and embedded in 812 resin (Spi-Chem) after gradient infiltration. Ultra-thin sections were cut using ultramicrotome (Leica UC7, Vienna, Austria) and double-stained by uranyl acetate and lead citrate. The ultrastructure of tissue was observed by TEM (HT7700, Japan).

### ATG5 knockdown experiment

HCC cells (SMMC7721, Huh7, H22) were seeded at density of 10^6^ cells per well in 6-well plates and transfected with 2500 ng ATG5 plasmid. Knockdown of ATG5 protein was confirmed by western blot with ATG5 antibody (1:1000).

### In vitro detection of ICD

First, HCC cells and ATG5-knockdown HCC cells were seeded into TC-treated cell slides and treated with drugs and heat stress. Next, the prepared cell slides were incubated with primary antibodies (anti-Calreticulin 1:100, anti-HMGB1 1:100) at 4 °C overnight, which then were incubated with fluorescent secondary antibodies (Alexa Fluor^®^ 488 1:500, Alexa Fluor^®^ 555 1:500) and DAPI stain for 1 h. Lastly, the expression of CRT and HMGB1 in cells was examined by CLSM. Additionally, the processed cells and supernatant were obtained. The ATP concentration in the cells and supernatant were measured by an ATP assay kit (Beyotime, China), and the concentration of HMGB1 in the supernatant was examined using an ELISA kit (Solarbio, Beijing). Bone marrow-derived dendritic cells (BMDCs) of mice were cultured with sublethally-heated HCC cells treated with different groups (PBS, ZIF-8, STF, SZP, SZP + shATG5) for 48 h. The collected DCs were stained by FITC anti-mouse CD11c, PE anti-mouse CD86, and APC anti-mouse CD80 antibodies (Biolegend), and subsequently measured via flow cytometry. ELISA kit was employed to identify the concentration of IL-12p70 (Lianke Bio, China EK212/3), IL1-β (Thermo Fisher Scientific 88-7013-88), IL-6 (Thermo Fisher Scientific 88-7064-88), and TNFa (Thermo Fisher Scientific 88-7324-88) in the supernatant of DCs.

### In vivo detection of ICD

H22 cells with ATG5 knocked-down and H22 cells were subcutaneously inoculated into the right thigh of BALB/c mice, respectively. Subsequently, IRFA mice model was established following the method described in Section “IRFA subcutaneous tumor model”. After four times tail vein injections of NPs, tumor tissues and tumor-draining lymph nodes were collected. Next, tumor tissue slides were incubated with primary antibodies (anti-CRT, anti-HMGB1) at 4 °C overnight and observed through CLSM; lymph nodes were examined via flow cytometry, following the method described in Section “In vitro detection of ICD”.

### In vivo vaccine inoculation trials

Firstly, a whole tumor cell vaccine was prepared. H22 cells were treated in three ways: heat, heat with STF (10 μM), and heat with SZP (10 μM), all of which were performed at 47 °C for 15 min. The cells were harvested after 24 h and trypan blue ensured cell death rate was higher than 80%. Next, 3 × 10^4^ dying cells were injected into the left back of BALB/c mice twice (n = 10), 7 d apart. Then, 14 days after the first injection, 5 × 10^5^ of H22 cells were subcutaneously injected into the contralateral thigh. On day 15, the spleen of the mice was collected and stained with APC/Fire™ 750 anti-mouse CD8a, PE anti-mouse CD44, and APC anti-mouse CD62L antibodies, which then were analyzed by flow cytometry. Tumors were measured with a caliper every 2–3 days when they grew visible. The mice were euthanized by inhaling CO_2_ on day 24. Meanwhile, ELISA kit was employed to measure the concentration of IFN-γ (Lianke Bio, China EK212/3) and TNF-α in the serum of mice.

### In vivo anti-tumor treatment and immune activation

Antitumor studies were carried out in H22 IRFA subcutaneous tumor model. As shown in Fig. 8A, 25 mice were categorized into five groups: control, STF, BZP, SZP, and SBZP. All drugs were injected 2 days before IRFA six times with interval of 2 days (STF: 8 mg/Kg; BMS: 4 mg/Kg). Since the 10th day after IRFA, the tumors were measured by caliper every 2 days, and the mice were weighed. On day 24, the tumor tissues were harvested after the final experiment. At the same time, ELISA kit was employed to measure the concentration of IFN-γ, TGF-β (Lianke Bio EK981-96), IL-6, and TNF-α in the serum of mice from different groups.

### Immunocytochemistry and immunofluorescence staining

Prepared tumor tissue sections were stained with Ki-67, TUNEL (One Step TUNEL Apoptosis Assay Kit, Beyotime), LC3B, P62, CD4, and CD8, respectively. Antibody concentrations used were all 1:100 for primary antibodies and 1:500 for secondary antibodies. Next, immunofluorescence and immunohistochemical analysis were carried out.

### Flow cytometry

The tumors and spleens of mice were digested into single-cell suspensions. The collected cells were divided into two groups: one was incubated and stained with PerCP anti-mouse CD45 (Biolegend 103,129), PE anti-mouse CD4 (Biolegend 100,407), and APC/Fire™ 750 anti-mouse CD8a Antibodies (Biolegend 100,765); the other was incubated and stained with PerCP anti-mouse CD45 (Biolegend 103,129), FITC anti-mouse CD11c (Biolegend 117,305), PE/Cyanine7 anti-mouse F4/80 (Biolegend 123,113), and PE anti-mouse CD11b Antibodies (CST#24,965). The cells were examined by flow cytometry and FlowJo was used for data analysis.

### Statistical analysis

Measurement data were expressed as mean ± standard deviation. Kaplan–Meier survival curves were compared using the log-rank Mantel–Cox test. Data were analyzed by GraphPad Prism 7.0 (GraphPad Software, San Diego, CA). P < 0.05 was considered significant.

## Results and discussion

### IRFA promotes autophagy in HCC cells and the emergence of an immunosuppressive microenvironment

**Fig. 1:**
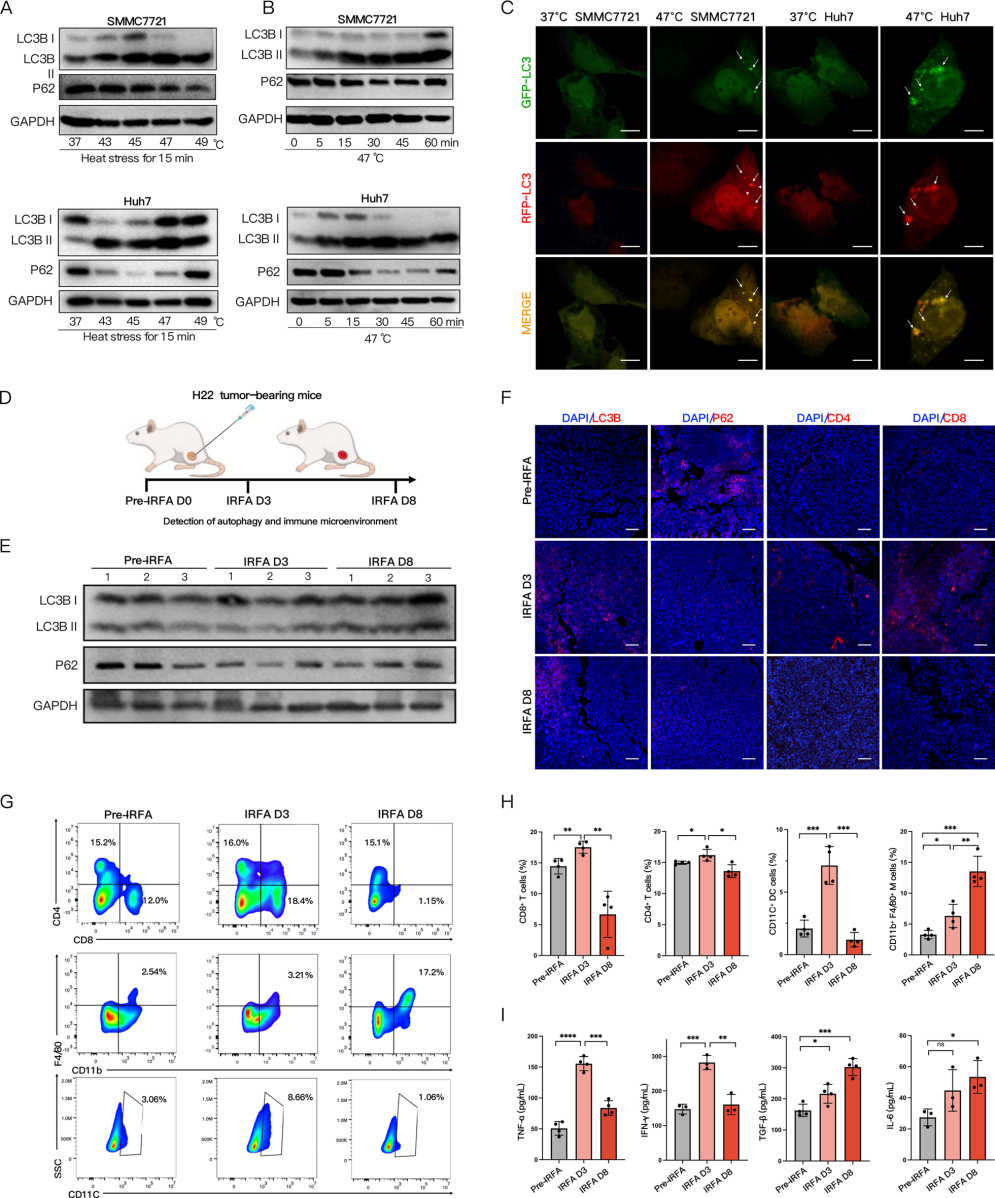
IRFA for HCC cells promotes autophagy with the emergence of an immunosuppressive microenvironment. **A** Western blot of LC3B and P62 after SMMC7721 and Huh7 cells heated at different temperatures (43, 45, 47, and 49 °C) for 15 min. **B** Immunoblotting of LC3B and P62 in SMMC7721 and Huh7 cells heated at 47 °C for different periods of time (0, 5, 15, 30, 45, and 60 min). **C** Representative CLSM images of SMMC7721 and Huh7 cells transfected with mRFP-eGFP–LC3 plasmid under heat stress (arrow pointing towards green spots, red spots, and yellow spots). Scale: 20 µm. **D** Schematic diagram of the establishment of IRFA subcutaneous HCC model. **E** Immunoblotting of LC3B and P62 in tumors before, 3 days, and 8 days after IRFA. **F** Representative CLSM images of LC3B, P62, CD4, and CD8 in tumors before, 3 days, and 8 days after IRFA. Scale: 100 µm. **G**, **H** Representative flow cytometry plots and quantitative assessments of CD4^+^ T cells, CD8^+^ T cells, CD11c^+^DCs and CD11b^+^/F4/80^+^ TAMs (of CD45^ +^ gate) in tumors before, 3 days, and 8 days after IRFA (n = 3). **I** Quantification of TNF-α, IFN-γ, TGF-β, IL-6 in mice serum before, 3 days, and 8 days after IRFA by ELISA (n = 4). The data were expressed as mean ± standard deviation. *p < 0.05, **p < 0.01, ***p < 0.001 and ****p < 0.0001. IRFA, Incomplete Radio Frequency Ablation; CLSM, Confocal laser scanning microscope; DCs, Dendritic cells; TAMs, Tumor-associated macrophages; ELISA, Enzyme-Linked Immunosorbent Assay

Previous research has revealed that IRFA can induce HCC cells’ resistance to heat treatment through protective autophagy [24, 42]. To explore the effects of thermal ablation on the autophagy of HCC cells, we assessed in vitro the autophagy profile of SMMC7721 and Huh7 cells exposed to heat stress at different temperatures (43, 45, 47, and 49 °C) for 15 min and at 47 °C for various periods of time (5, 10, 15, 30, and 60 min). The western blot results demonstrated that the autophagy level of SMMC7721 and Huh7 cells was positively correlated to temperature and the duration of heat treatment, featured by an increased conversion of LC3-I to LC3-II and a decreased expression of P62. Autophagy activation was most prominent in HCC cells heated at 47 °C for 15 min (Fig. 1A, B). “Autophagic phenotype” of “vacuoles” in the cytoplasm was found in sublethally heated SMMC7721 and Huh7 cells via microscope (Additional file 1: Fig. S1). Therefore, the condition of HCC cells being heated at 47 °C for 15 min was determined as the optimal method to activate autophagy in subsequent experiments, which was consistent with other results [43, 44]. Then we transfected transiently SMMC7721 and Huh7 cells with mRFP-GFP-LC3 plasmid to observe the changes in autophagic flux after sublethal heat stress. When autophagosomes fused with lysosomes, GFP in mRFP-GFP-LC3 was quenched in the acidic environment of autolysosome, with the red fluorescence emitted from mRFP left. Thus, autolysosomes appeared as red dots (only mRFP) while autophagosomes as yellow ones (merged of mRFP and GFP). CLSM showed a significantly increased number of red and yellow spots after sublethal heat stress (Fig. 1C and Additional file 1: Fig. S8), with red spots outnumbering yellow ones. This suggested that sublethal heat stress could induce more autophagosomes in HCC cells without impeding autophagic flux. Furthermore, we examined how IRFA influenced the autophagy levels in H22-bearing mice via western blot (Fig. 1E). The results showed that the autophagy level was prominently elevated 3 days and 8 days after IRFA compared with that before IRFA. Immunofluorescent staining and Immunohistochemical results also exhibited the same changes (Fig. 1F and Additional file 1: Fig. S2).

To further investigate the changes in the tumor immune microenvironment after IRFA, we analyzed the proportion of tumor-infiltrating CD4^+^ and CD8^+^T cells by flow cytometry (Fig. 1G), immunohistochemistry (Additional file 1: Fig. S5), and immunofluorescence (Fig. 1F). It turned out that the infiltration of CD4^+^ and CD8^+^T cells in the tumor tissue increased 3 days after IRFA, but dropped 8 days after IRFA (Fig. 1H). At the same time, the tumor-infiltrating CD11C^+^ DCs evaluated by flow cytometry suggested trends consistent with that of T cells. On top of that, we discovered that 3 and 8 days after IRFA, the infiltration rate of CD11b^+^F4/80^+^ TAMs continuously grew to 1.92 ± 0.50 and 4.12 ± 0.64 folds of pre-IRFA levels, respectively. Similar changes could also be identified from the ratio of immune cells in the spleen of the mice (Additional file 1: Fig. S3, S4). According to ELISA, the levels of tumor-killing cytokines (TNF-α, IFN-γ) shot up on the third day after IRFA, but plummeted 8 days after IRFA; meanwhile, the levels of tumor-promoting cytokines (TGF-β, IL-6) rose progressively (Fig. 1I). These results suggested that the immune response was mildly activated 3 days after IRFA, which could be linked to inflammatory cell infiltration caused by IRFA. Yet such immune activation was transient, which was followed by the emergence of a prominently suppressive immune microenvironment 8 days after IRFA.

### Synthesis and characterization of SBZP

**Scheme 1:**
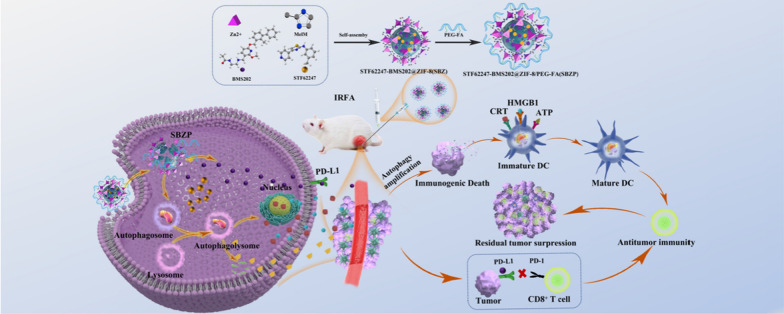
The preparation of STF-BMS@ZIF-8/PEG-FA; Autophagic nanoparticles evoke ICD combined with anti-PD-1/PDL1 therapy to remodel the immune microenvironment after IRFA

**Fig. 2:**
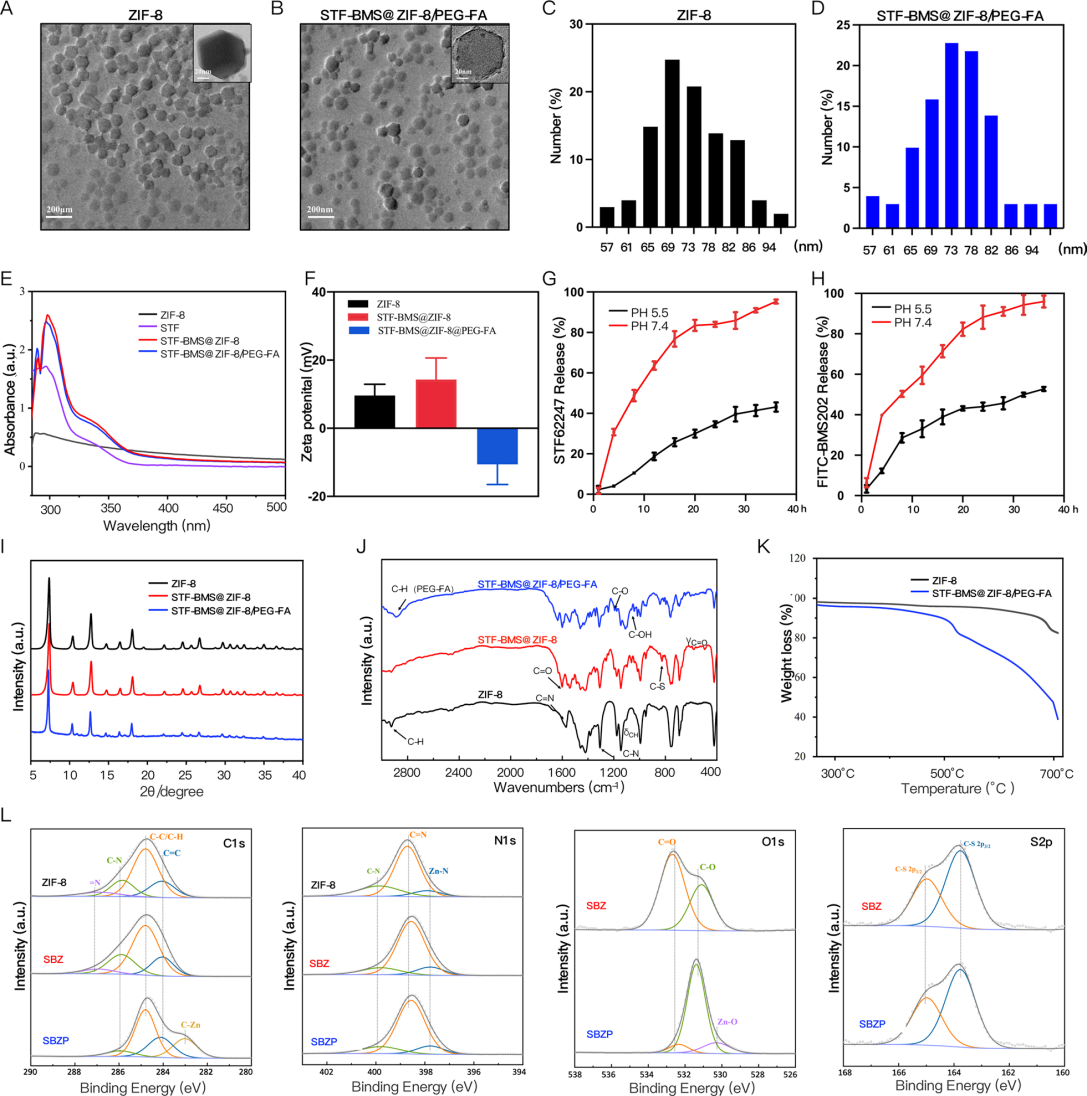
Preparation and Characterization of STF-BMS@ZIF-8/PEG-FA. **A**, **B** TEM images of ZIF-8 and STF-BMS@ZIF-8/PEG-FA. Scale: 20 nm. **C**, **D** TEM test the particle size distribution of ZIF-8 and STF-BMS@ZIF-8/PEG-FA. **E** Ultraviolet–visible spectroscopy of ZIF-8, STF, STF-BMS@ZIF-8, STF-BMS@ZIF-8/PEG-FA. **F** Zeta potentials of ZIF-8, STF-BMS@ZIF-8, and STF-BMS@ZIF-8/PEG-FA (n = 3). **G**, **H** pH-responsive release percentage of STF and BMS from STF-BMS@ZIF8/PEG-FA in different buffers at pH 5.5 and 7.4.** I** XRD of ZIF-8, STF-BMS@ZIF-8, and STF-BMS@ZIF-8/PEG-FA. **J** FITR of ZIF-8, STF-BMS@ZIF-8, and STF-BMS@ZIF-8/PEG-FA. **K** Thermogravimetric analysis of ZIF-8 and STF-BMS@ZIF-8/PEG-FA. **L** XPS spectra of ZIF-8, STF-BMS@ZIF-8, and STF-BMS@ZIF-8/PEG-FA (C1s, N1s, O1s, S2p). ZIF-8, zeolitic imidazolate framework-8; STF, STF62247; BMS202, BMS; SBZ, STF- BMS@ZIF-8; SBZP, STF- BMS @ZIF-8@PEG-FA; TEM, Transmission emission micrography; FTIR, Fourier transform IR; XRD, X-ray diffraction; XPS, X-ray photoelectron spectroscopy; FITC, Fluorescein Isothiocyanate

Previous research unraveled the close connection between autophagy and TME. Exploring methods for modulating autophagy could reverse the immunosuppressive microenvironment and facilitate immunotherapy [12, 45–47]. STF can potently induce ACD. On top of that, BMS inhibits the interaction of PD-1/PD-L1 by binding to PD-L1 and causing PD-L1 dimerization. Therefore, we loaded STF and BMS into the ZIF-8 framework to synthesize SBZP NPs through “one-pot synthesis”. The detailed procedure of preparation was illustrated in Scheme 1. The morphology of ZIF-8 and SBZP was observed by TEM. It turned out that a polyhedral ZIF-8 nanocarrier was successfully constructed (Fig. 2A, B). The diameter of SBZP NPs was larger than that of ZIF-8 after it was loaded with STF, BMS, and PEG-FA, which could be attributed to the drug coating’s modification in the surface of NPs (Fig. 2C, D). The characteristic ultraviolet absorption peak (about 294 nm) of STF in SBZP was detected in the UV scanning spectrum, indicating the successful loading of STF. Since the characteristic absorption peak of BMS (about 220 nm) almost coincided with that of ZIF-8, it was hard to detect the absorption peak of BMS (Fig. 2E). Zeta potential of ZIF-8, SBZ, and SBZP detected by dynamic light scattering (DLS) was 9.62 ± 3.29 mV, 14.3 ± 6.3 mV, –10.9 ± 10.9 mV, respectively (Fig. 2F). Such variation was due to the slightly positive charge in STF and BMS, and the encapsulation of the negatively charged [–]COO–PEG–COO[–]. XRD verified that SBZP was highly consistent with the ZIF-8 nanocarrier, indicating that the structure of ZIF-8 underwent no significant change after modification. However, the XRD peaks of SBZP modified by PEG-FA were broadened to some extent (Fig. 2I). The characteristic absorption peaks of ZIF-8, SBZ, and SBZP under different spectra were compared by FTIR. In the spectrum of ZIF-8, the bands at 2930 cm^−1^, 1570 cm^−1^, 1308 cm^−1^, 848 cm^−1^ and 756 cm^−1^ corresponded to C-H stretching, C = N stretching, C-N stretching, and CH bending. In the spectrum of SBZ, the band at 1630 cm^−1^ corresponded to C = O stretching, while those at 578 and 496 cm^−1^ corresponded to C = O bending, indicating that BMS was successfully loaded into ZIF-8. The band at 826 cm^−1^ corresponding to C-S stretching suggested that STF was successfully loaded into ZIF-8. In the spectrum of SBZP, bands at 2890 cm^−1^, 1240^–1^, and 1110 cm^−1^ corresponded to the introduction of C-H, C-O, and C–OH, respectively, indicating the successful modification of PEG-FA (Fig. 2J). Thermal profile analysis of SBZP implied the encapsulation of STF, BMS, and PEG-FA in ZIF-8 (Fig. 2K). XPS was used to identify changes in chemical composition (Fig. 2L). In comparison with ZIF-8, C-O (531.1 eV, 36.39 at%), C = O (532.69 eV, 63.61 at%), C-S bond (163.77/164.98 eV) were detected in SBPZ, which proved the successful loading of STF and BMS. Meanwhile, the appearance of new bonds such as Zn–O (530.3 eV) and Zn-C (283.02 eV) in SBZP signified the binding of PEG-FA. In addition, the pH-sensitive release of STF and BMS from SBZP at pH 5.5 and pH 7.4 was further studied. The results showed that PBS (pH 7.4) resulted in only a small amount of STF and BMS released. In contrast, 24 h after exposure to an acidic environment (pH 5.5), SBZP released 84.04 ± 1.31 % STF and 88.21 ± 5.78 % BMS (Fig. 2G, H). Such excellent pH-dependent release properties of SBZP qualified it as an ideal carrier for delivering tumor drugs.

### Cellular uptake, biodistribution and cytotoxicity of ZIF-8/PEG-FA NPs

**Fig. 3:**
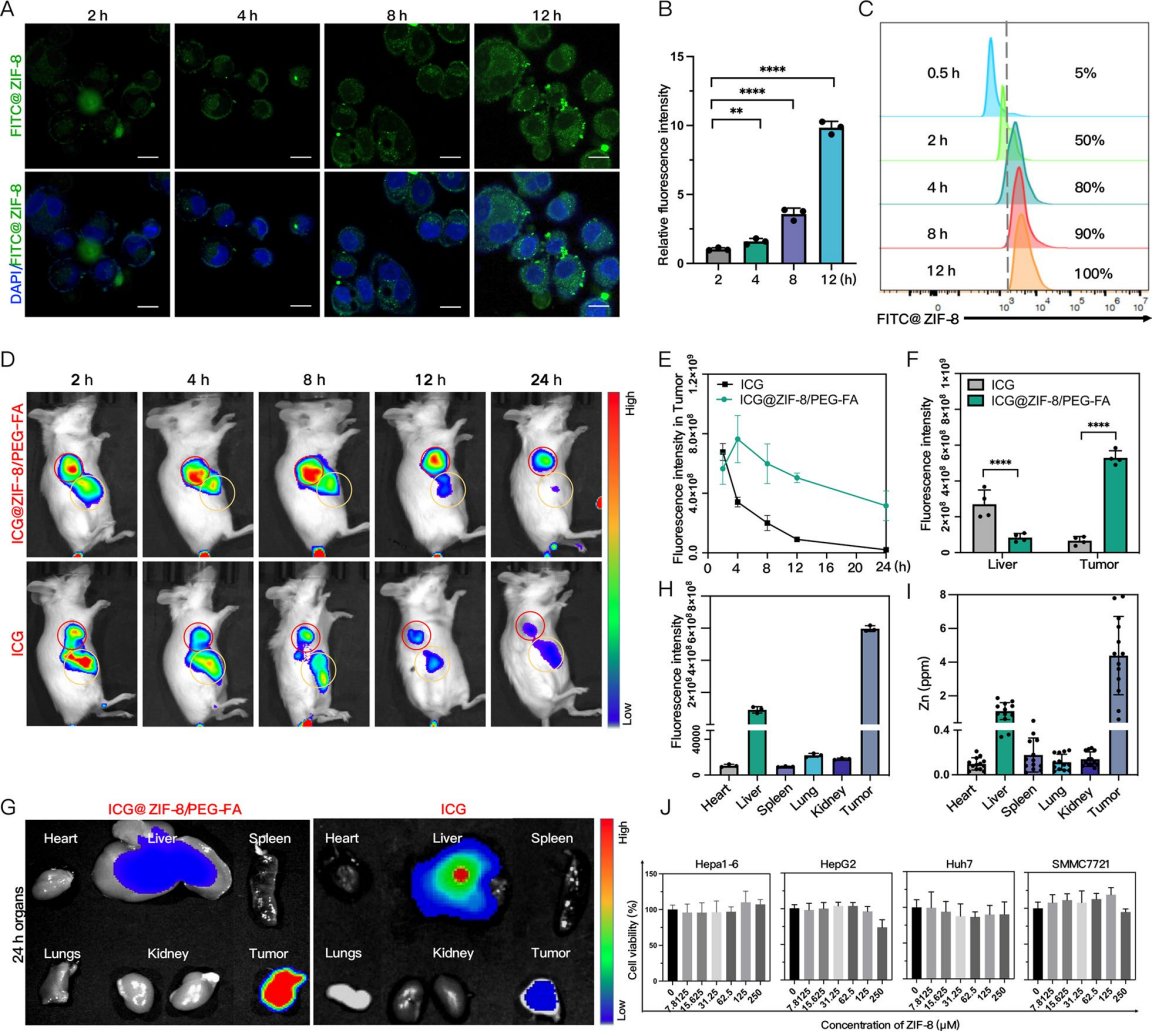
Cellular uptake, biodistribution and cytotoxicity of ZIF-8/PEG-FA NPs. **A**, **B** The cellular uptake and fluorescence quantitative analysis of SMMC7721 cells incubated with FITC@ZIF-8 for different periods of time via CLMS(n = 3). Scale: 20 µm. **C** Assessment of the ratio of SMMC7721 cells taken in by FITC via flow cytometry. **D**, **E** Representative fluorescence imaging (red circles indicate tumors, yellow circles indicate liver) and trend curve analysis of ICG@ZIF-8/PEG-FA NPs and free ICG in mice (n = 3). **F**, **G** Representative dissected liver and tumors fluorescence imaging and quantitative analysis of ICG@ZIF-8/PEG-FA NPs and free ICG (n = 4). **H** Dissected organs and tumors fluorescence quantitative analysis of ICG@ZIF-8/PEG-FA NPs (n = 4). **I** The content of Zn^2^^+^ in dissected organs and tumors evaluated by ICP-OES of ICG@ZIF-8/PEG-FA NPs (n = 8). **J** The viability of SMMC7721, Huh7, HEPG2, and Hepa1-6 cells after being incubated with ZIF-8 NPs of a specific concentration for 24 h (n = 3). Results were expressed as mean ± SD, *p < 0.05, **p < 0.01, ***p < 0.001, and ****p < 0.0001. ICG, Indocyanine Green; ICP-OES, Inductively Coupled Plasma Atomic Emission spectroscopy

To explore HCC cells’ uptake of the NPs, we successfully synthesized FITC-labeled ZIF-8. Immunofluorescence demonstrated that SMMC7721 cells could rapidly take up FITC@ZIF-8 NPs and the fluorescence intensity gradually magnified (Fig. 3A, B). Flow cytometry analysis demonstrated the increased accretion of SMMC7721 cells that had taken up FITC-labeled NPs over time (Fig. 3C) and 100% cellular uptake of NPs was detected 12 h after incubation.

Then we synthesized active targeting ZIF-8/PEG-FA NPs labeled by ICG and identified their biodistribution in H22-bearing mice compared with free ICG. The IVIS images (Fig. 3D) illustrated that free ICG quickly gathered in the liver and tumor tissues. Twelve to twenty-four hours later, the fluorescent intensity of free ICG reduced, and it was excreted from the liver rapidly. In comparison, ICG@ZIF-8@PEG-FA NPs accumulated in the tumor tissues longer. Their fluorescence signal in tumors peaked at 4 h and lasted at least 24 h (Fig. 3E). The quantitative fluorescence trend chart suggested that ICG@ZIF-8@PEG-FA NPs may possess better cycling stability and therefore increase drug accumulation in local tumors. Additionally, according to the fluorescence imaging of major organs and tumors ex vivo 24 h after injection, ICG@ZIF-8@PEG-FA NPs mainly accumulated in tumors and fractionally in the livers (Fig. 3F), while no obvious fluorescence signals were spotted in other organs (Fig. 3H). At the same time, the examination of Zn^2+^ accumulation in the tissues through ICP-OE also revealed consistent results (Fig. 3I). That ICG@ZIF-8@PEG-FA NPs could effectively accumulate in residual tumor tissues after IRFA may be ascribed to the enhanced EPR effect we previously reported [32] and the active targeting modification. Meanwhile, the CCK8 experiment discovered no apparent cytotoxicity of serial concentration of ZIF-8 NPs. The cell viability was above 90% even at a concentration of 250 µm (Fig. 3J).

### SZP enhances autophagy of sublethally heated HCC cells

**Fig. 4:**
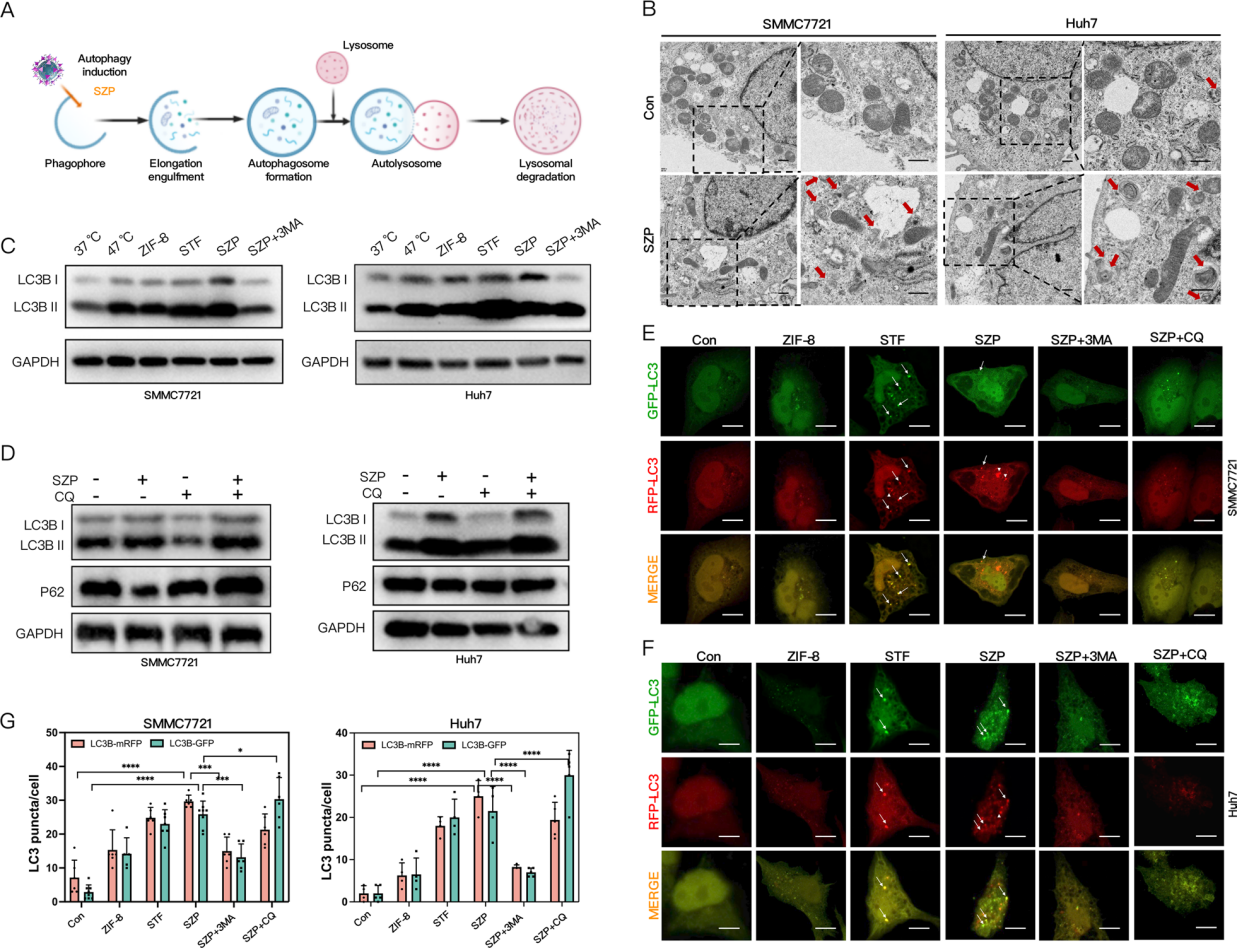
SZP enhances autophagy in sublethally heated HCC cells. **A** Diagram illustrating how autophagy was induced by SZP. **B** The ultrastructure of tumor cells after SZP NPs treatment observed via TEM, with autophagosomes and autolysosomes pointed out by arrows. Scale: 1 µm. **C** Immunoblotting of LC3B in SMMC7721 and Huh7 cells pre-treated with 3MA under sublethal heat stress caused by SZP. **D** Immunoblotting of LC3B and P62 in SMMC7721 and Huh7 cells pre-treated with CQ under sublethal heat stress induced by SZP. **E**–**G** Representative CLSM images and quantitative analysis of HCC cells transfected with mRFP-eGFP-LC3 plasmid under the influence of different groups (arrow pointing towards green spots, red spots, and yellow spots). Scale: 20 µm. Results were expressed as mean ± SD, *p < 0.05, **p < 0.01, ***p < 0.001, and ****p < 0.0001. SZP, STF62247@ZIF-8/PEG-FA; TEM, Transmission Electron Microscope; 3MA, 3-Methyladenine; CQ, Chloroquine

The process of autophagy can be divided into three relatively independent steps (Fig. 4A): the formation of autophagosome, the fusion of autophagosome and lysosome, namely autolysosome, and the degradation. Since sublethal heat stress induced protective autophagy of HCC cells, we further amplified autophagy via SZP which was loaded with STF to induce ACD [43–45] in residual tumor cells. To confirm the effect, we incubated sublethally heated SMMC7721 and Huh7 cells with SZP for 4 h. TEM detection revealed that SZP led to a marked increase of autophagosome-like double membrane vesicles in cytoplasm (Fig. 4B). Since autophagic flux is a dynamic process, we further verified whether the enhanced autophagy was attributed to the increased formation of autophagosome or the inhibition of its fusion with lysosome. As we know, 3-methyladenine (3-MA) could inhibit the formation of autophagosomes while Chloroquine (CQ) could inhibit the fusion of autolysosome, both resulting in autophagy inhibition. Western blot demonstrated that compared to the control group, SZP significantly promoted the transformation of LC3B-I to LC3B-II in SMMC7721 and Huh7 cells. It demonstrated that SZP further amplified autophagy induced by sublethal heat. At the same time, SZP failed to elicit such remarkable changes when the cells were pretreated with 3-MA (Fig. 4C). However, the expression of both LC3B and P62 was further elevated when the autophagic flux of SZP-treated cells was blocked by CQ (Fig. 4D). These results suggested that the SZP-induced increase in autophagic flux resulted from the incremented formation of autophagosomes. At the same time, we transfected cells with mRFP-GFP-LC3 plasmid to trace the changes in autophagic flux, and the same conclusion was reached. Immunofluorescent results visually revealed that after cells were treated with SZP, the number of autophagosome and autolysosomes multiplied more than 20 times compared with that of the control group (Fig. 4E, F). When CQ was added, the number of autolysosomes was reduced because of inhibited fusion of autolysosome, but autophagosomes continued to increase (Fig. 4G). In summary, the enhanced autophagy induced by SZP NPs in sublethally heated cells was ascribed to the formation of autophagosomes instead of the inhibition of autolysosome degradation. This ultimately led to the potent activation of autophagic flux, which was necessary for inducing ACD [48].

### SZP mediates ACD

**Fig. 5:**
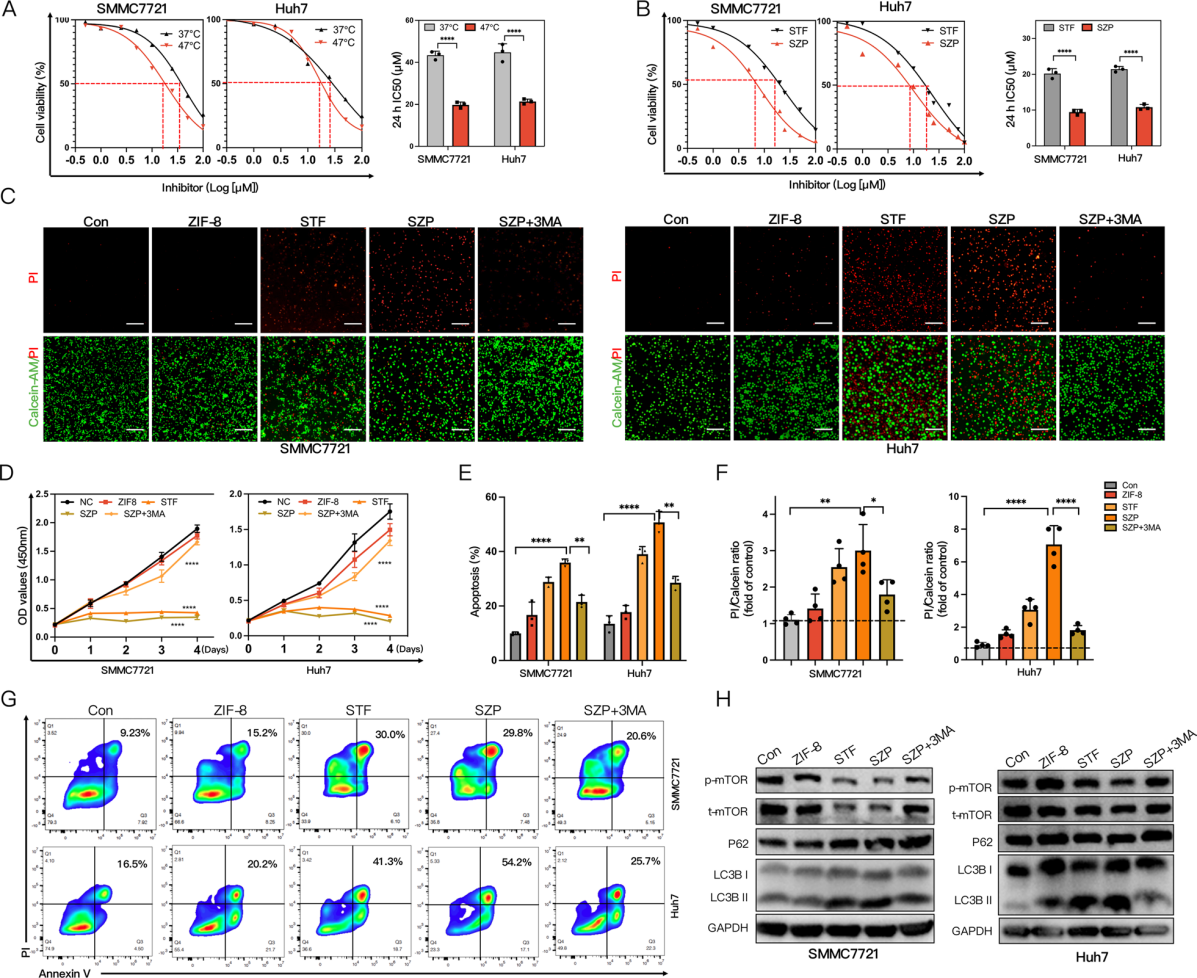
SZP mediates ACD. **A** The dose–response curve for CCK8 assay of IC50 of STF62247 in different concentrations treating cells at 37 °C and 47 °C for 24 h (n = 3). **B** The dose–response curve for CCK8 assay of IC50 of STF62247 and SZP NPs in different concentrations treating HCC cells at 47 °C for 24 h (n = 3). **C**, **F** Live/dead cells double staining and quantitative analysis of sublethally heated SMMC7721 and Huh7 cells exposed to different groups (n = 4). Scale: 1000 µm. **D** Analysis of the proliferation of sublethally heated SMMC7721 and Huh7 cells under influence of different groups by CCK8 (n = 3). **E**, **G** Representative flow cytometry plots and quantitative analysis of apoptosis in sublethally heated SMMC7721 and Huh7 cells under influence of different conditions (n = 3). **H** Immunoblotting of p-mTOR, t-mTOR, P62, and LC3B in sublethally heated SMMC7721 and Huh7 cells under influence of different groups. The results were expressed as mean ± SD, *p < 0.05, **p < 0.01, ***p < 0.001, and ****p < 0.0001. ACD, Autophagy-dependent cell death; IC50, Half maximal inhibitory concentration

Next, we investigated whether SZP could induce ACD in sublethally heated HCC cells. HCC cells and sublethally heated cells were treated with different concentrations of STF. According to the cell viability curve, the 24 h half maximal inhibitory concentration (IC50) of STF at 37 °C was 2.41 (SMMC7721) and 2.12 times (Huh7) of that at 47 °C (Fig. 5A). Similar results were acquired for the comparison of 48 h IC50: STF’s IC50 at 37 °C was 2.78 (SMMC7721) and 2.27 times (Huh7) of that at 47 °C (Additional file 1: Fig. S6). The results suggested that STF substantially enlarged the effect of sublethal heat stress on killing HCC cells. We also compared the impact of STF and SZP on the cell viability of sublethally heated HCC cells. It turned out that the 24 h IC50 of STF in SMMC7721 and Huh7 cells was 1.92 and 1.83 times that of SZP (Fig. 5B), and the comparison of 48 h IC50 yielded similar results (Additional file 1: Fig. S7), indicating that SZP outperformed STF in suppressing the survival of sublethally heated HCC cells. In summary, sublethal heat stress activated low-level autophagy and ACD could be induced by further amplified autophagy activation. Importantly, sublethally heated HCC cells exhibited higher sensitivity to this strategy than normal ones. The SZP NPs we prepared outmatched STF in inducing ACD.

We continued to verify whether the SZP-mediated cell death in residual tumors relied on autophagy. SMMC7721 and Huh7 cells were incubated with ZIF-8, STF, SZP, and SZP + 3MA for 4 h, respectively. Then, they were exposed to sublethal heat stress at 47 °C. After 24 h, CCK8 assay (Fig. 5D), live/dead cell staining (Fig. 5C), apoptosis experiment (Fig. 5E, G), and colony formation assay (Additional file 1: Fig. S11, S12) were carried out. The results pointed out the ability of SZP to inhibit cell proliferation, promote cell death, and induce cell apoptosis. We also found that the killing effect induced by SZP on cells could be obviously attenuated by 3-MA pretreatment. It has been widely recognized that mTOR signaling pathway participated in the suppression of autophagy. In this research, western blot uncovered that both STF and SZP could significantly suppress the expression of p-mTOR protein of sublethally heated SMMC7721 and Huh7 cells in an autophagy-dependent manner, which is proved by the co-treatment of SZP and 3-MA (Fig. 5H). In conclusion, SZP NPs suppressed the proliferation of tumor cells as well as enhanced ACD by inhibiting mTOR signaling pathway.

### SZP induces autophagy-dependent ICD in vitro

**Fig. 6:**
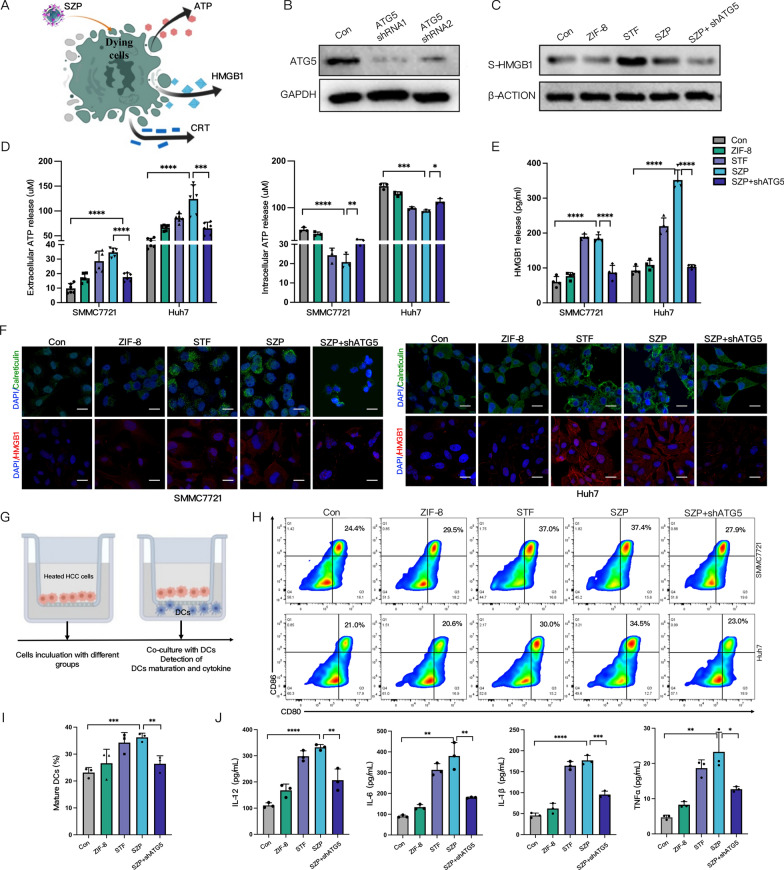
SZP induces autophagy-dependent ICD in vitro. **A** Schematic diagram of SZP inducing ICD. **B** Reduced expression of ATG5 in HCC cells with ATG5 knocked down. **C**, **E** Immunoblotting and ELISA of HMGB1 in the supernatant of sublethally heated HCC cells (SMMC7721 and Huh7) and ATG5-knockdown HCC cells treated with SZP (n = 4). **D** The concentration of ATP within and outside the sublethally heated HCC cells and ATG5-knockdown HCC cells treated with SZP (n = 5). **F** Representative CLSM graphs of the CRT exposure and HMGB1 release of sublethally heated HCC cells and ATG5-knockdown HCC cells treated with SZP. Scale: 30 µm. **G** Schematic diagram of transwell co-culture. Sublethally heated HCC cells and ATG5-knockdown HCC cells treated with SZP (upper chamber) and BMDCs (lower chamber) were co-cultured for 24 h. **H**, **I** Representative flow cytometry plots and quantitative analysis of the maturation of DCs (of CD11C^+^ gate) (n = 3). **J** The levels of IL-12p70, IL-1β, TGF-β, and IL-6 in the supernatants of co-cultured DCs measured by ELISA (n = 4). Results were expressed as mean ± SD, *p < 0.05, **p < 0.01, ***p < 0.001, and ****p < 0.0001. ICD, Immunogenic death; CRT, Calreticulin; BMDCs, Bone marrow-derived DC cells

Previous studies have suggested that autophagy plays a pivotal role in ICD for its involvement in the release of DAMP from dead cells [49]. Calreticulin (CRT) exposure, HMGB1 release, and ATP secretion signified the occurrence of ICD (Fig. 6A). Therefore, we examined whether SZP could evoke the ICD of sublethally heated HCC cells via autophagy. ATG5 is recognized as a key protein in autophagy, and the knockdown of ATG5 causes STF unable to induce autophagy [43]. To analyze the role of SZP-induced autophagy in ICD, we first established cell lines (SMMC7721, Huh7, and H22 cells) with the knockdown of ATG5. Western blot confirmed that the expression of ATG5 was diminished in the cells transfected with ATG5 shRNA (Fig. 6B). CRT exposure was an initial event in ICD cascade that served as an activator of immune cells, HMGB1 mainly acted as an immunogenic mediator in the tumor, and extracellular release of ATP triggered DCs activation. Firstly, we used immunofluorescence staining (Fig. 6F and Additional file 2: Fig. S14) and flow cytometry (Additional file 2: Fig. S13) to explore the CRT exposure levels in sublethally heated SMMC7721 and Huh7 cells that were treated with SZP. The results demonstrated that SZP significantly reinforced CRT exposure. HMGB1 was a highly abundant chromatin-binding protein. According to the immunofluorescent results, HMGB1, stimulated by SZP-induced autophagy, translocated from nucleus to cytosol (Fig. 6F). ELISA (Fig. 6E) and Western blot (Fig. 6C) were employed to further quantify the levels of HMGB1 released to the supernatant of sublethally heated cells treated with SZP. The results indicated that SZP prominently potentiated the release of HMGB1. Next, the intracellular and extracellular concentrations of ATP were evaluated. We noticed that ATP secretion rose by 3.7-fold after sublethally heated cells underwent treatment of SZP, while the intracellular concentration of ATP declined visibly (Fig. 6D). However, a considerable drop in the expression and release of immunogenic molecules was detected after ATG5 knockdown in the above situation. It was concluded that SZP NPs could evoke the ICD of sublethally heated HCC cells in an autophagy-dependent manner.

The DAMP generated from ICD held the key to the maturation of DCs. Accordingly, we evaluated the effect of SZP in boosting the maturation of DCs. BMDCs were co-cultured with sublethally heated SMMC7721 and Huh7 cells treated with different groups (Fig. 6G). The results of flow cytometry revealed that the ratio of mature DCs (CD11c ^+^ CD80 ^+^ CD86 ^+^) significantly increased in the group treated with SZP to 2.3 times that of the control group, indicating that SZP boost the maturation of DCs. However, the group with ATG5 knockdown reversed the maturation of DCs induced by SZP (Fig. 6H, I). Additionally, elevated secretion of cytokines (IL-12p70, IL-10, TGF-β, and IL-6) that embody DCs activation (Fig. 6J) was detected by ELISA. In summary, SZP NPs could evoke ICD of sublethally heated HCC cells in an autophagy-dependent manner and boosted the maturation of DCs.

### Autophagy-dependent ICD induced by SZP and immune vaccine experiments in vivo

**Fig. 7:**
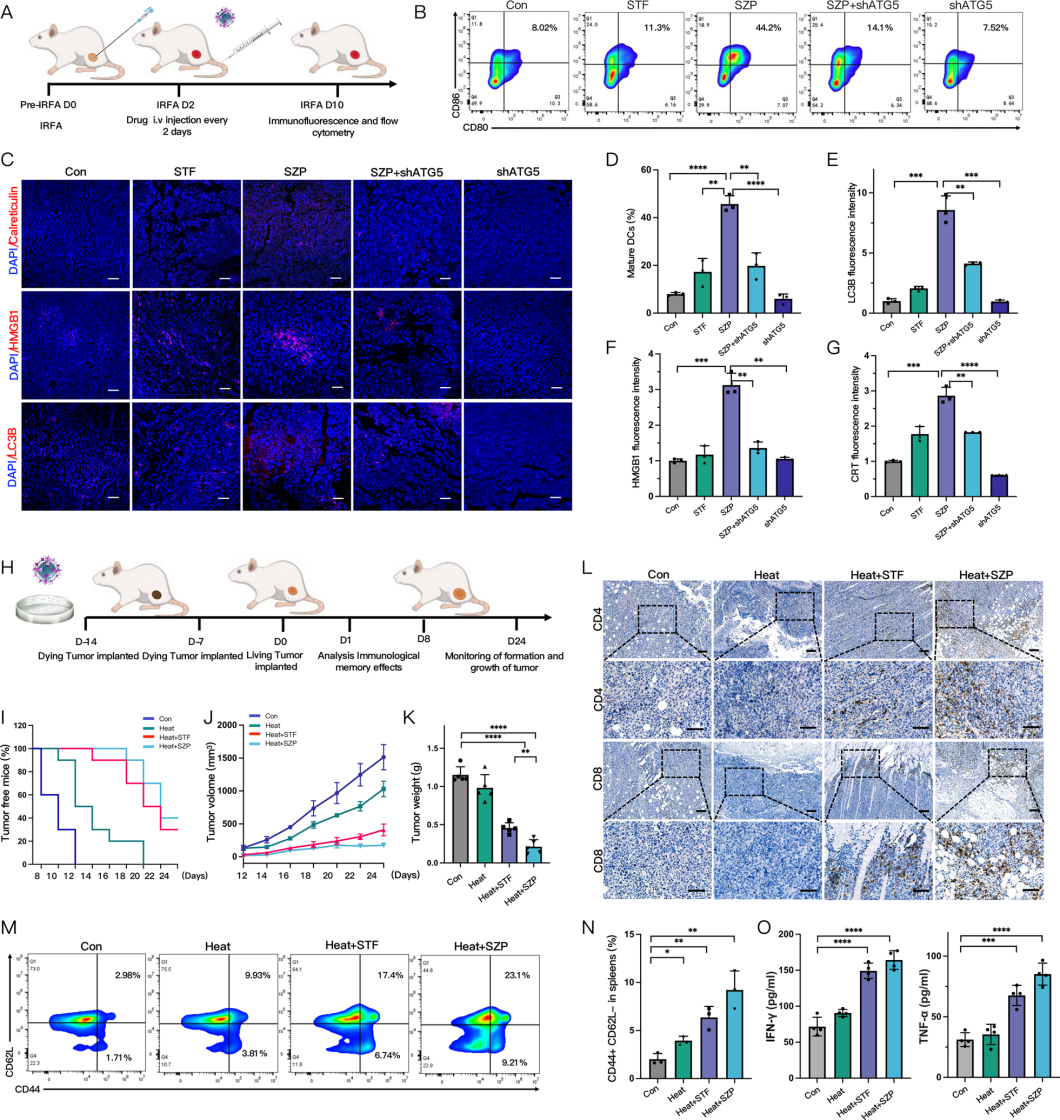
Autophagy-dependent ICD induced by SZP and immune vaccine experiments in vivo. **A** Flow chart of in vivo detection of ICD in tumor-bearing mice. **B**, **D** Detection and quantitative analysis of mature DCs (of CD11C^+^ gate) in tumor-draining lymph nodes via flow cytometry (n = 3). **C**, **E**–**G** Expression and quantitative analysis of CRT, HMGB1, and LC3B in tumors via CLSM (n = 3). **H** Flow chart of in vivo inoculation with immune vaccine. **I**: Kaplan–Meier plot of survival rate in different groups of tumor-free mice (n = 10). **J**, **K** Comparison of tumor growth curves and tumor weights in mice of different groups (n = 6). **L** Representative IHC images showing the expression of CD4 and CD8 in mice tumor of different groups. **M**, **N** Representative flow cytometry plots and quantitative analysis of TEM (of CD8^+^ gate) in the spleen of mice (n = 3). **O** The levels of IFN-γ and TNF-α in mice serum from inoculated with different immune vaccines by ELISA (n = 4). Results were expressed as mean ± SD, *p < 0.05, **p < 0.01, ***p < 0.001, and ****p < 0.0001. IHC, Immunohistochemistry; TEM, effector memory T cells

We established the IRFA subcutaneous H22 model to further evaluate whether SZP could induce ICD in vivo. SZP NPs (concentration of STF: 4 mg/kg) were injected through the tail vein every 2 days, 4 consecutive times (Fig. 7A). The release of DAMP which characterized ICD was investigated. Immunofluorescent analysis showed that SZP increased CRT exposure, HMGB1 release as well as LC3 expression in tumor tissues (Fig. 7C). However, in ATG5-knockdown mice tumors, SZP-induced DAMP release was significantly reduced (Fig. 7E, F, G). Meanwhile, we collected tumor-draining lymph nodes (TDLN) of the mice for evaluating DCs maturation. It was found that SZP treatment significantly promoted the maturation of DCs, which was 2.5-fold higher than that of the control group. On the contrary, SZP treatment failed to induce DC maturation in ATG5-knockdown mice tumors (Fig. 7B, D). These results illustrated that SZP NPs triggered ICD in residual tumors after IRFA and then contributed to the maturation of DCs in an autophagy-dependence manner.

To investigate whether SZP could activate an effective anti-tumor immune response after IRFA, we carried out a vaccination experiment, which is the gold standard for evaluating ICD in vivo. Firstly, H22 cells were exposed to different treatments (sublethal heat, sublethal heat + STF, sublethal heat + SZP) for 24 h as a tumor cell vaccine and injected into the left back of BALB/c mice twice with an interval of 7 days (Fig. 7H). Subsequently, the contralateral side of the mice was challenged with live H22 cells. It turned out that 12 days after tumor transplantation, new tumors could be visibly noticed in the control group and the heat group, but tumors in the latter group grew slower than those in the former. Such difference implied that heat-treated cell vaccine was a weak ICD-inducer that could not prominently suppress the growth of new tumors. On day 24, vaccination with heat + STF-treated cells and heat + SZP-treated cells significantly suppressed the growth of new tumors with a 30% (3/10) and 40% (4/10) tumor-free ratio, respectively (Fig. 7I). This suggested that vaccination derived from heat + STF and heat + SZP played a strong role in impeding tumor initiation. Moreover, the tumor grew much more slowly in the 6 tumor-developed mice of the heat + SZP group (Fig. 7J). Compared to the control group, the inhibition rates of tumor growth in the heat + SZP group and heat + STF group were 81.55 ± 5.96% and 61.55 ± 5.96% accordingly (Fig. 7K). It implied that SZP may serve as a more potent ICD-inducer of sublethally heated cells. Meanwhile, immunohistochemical results revealed that SZP could activate the systemic anti-tumor immune response, which was verified by the substantial increase in CD4^+^ and CD8^+^ T cell infiltration in tumor tissues (Fig. 7L). A significant rise in the secretion of IFN-γ and TNF-α (Fig. 7O) was also captured, the former of which was a typical anti-tumor cytokine secreted from activated T cells, and the latter was a cytokine from macrophage that could directly kill tumors and mediate immunity [50]. Furthermore, we collected the spleens of mice after the injection of H22 cells and analyzed the effector memory T cell (TEM) in the spleen via flow cytometry. The results demonstrated that there was an obviously higher percentage of TEM in the spleen undergoing heat + STF-treated and heat + SZP-treated cells vaccine (Fig. 7M), which was 4.58 and 3.16 times as that of the control group (Fig. 7N). In conclusion, it was considered that vaccination with heat + SZP-treated cells could act as in situ vaccine to suppress the growth of new tumors, activate a systemic anti-tumor immune response, and establish long-term immunological memory.

### SBZP inhibits the growth of residual tumors after IRFA

**Fig. 8:**
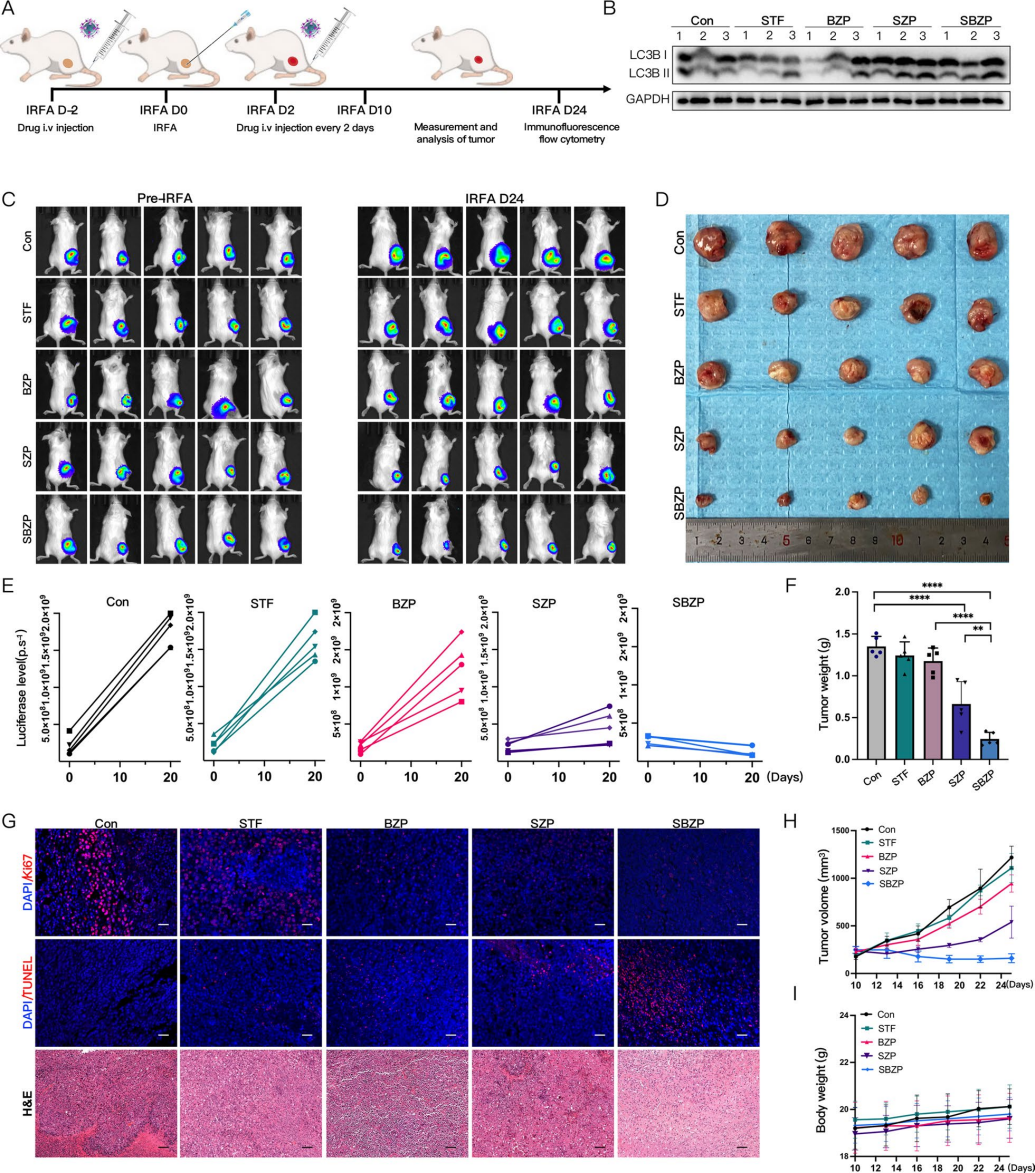
SBZP inhibits the growth of residual tumors after IRFA. **A** Flow chart of in vivo IRFA for subcutaneous tumors with the treatment of NPs. **B** Immunoblotting of LC3B in tumor tissues treated with different NPs. **C**, **E** IVIS bioluminescent imaging and quantitative analysis of mice tumors before and 24 days after IRFA (n = 5). **D**, **F**, **H**, **I** Photos, weights, and volumes of the tumors in mice were treated with different NPs (n = 5). **G** Representative CLSM graphs of TUNEL, Ki-67 fluorescent staining, and H&E of tumor tissues treated with different NPs. Scale: 100 µm. Results were expressed as mean ± SD, *p*p* < 0.05, **p < 0.01, ***p < 0.001, and ****p < 0.0001. IVIS, In vivo Imaging System

Encouraged by the results of activating ICD and establishing immune memory in vivo, we further evaluated whether SZP could reverse the immunosuppressive microenvironment after IRFA and thus solve the clinical difficulty of poor sensitivity to anti-PD-1/PD-L1 therapy. BMS is a novel small-molecule inhibitor, characterized by high oral bioavailability, strong permeability into solid tumors, and low cost compared with anti-PD-1/PD-L1 antibodies. However, features such as aggregation and hydrophobicity in aqueous media limit its efficacy, which can be overcome by the encapsulation of nanocarrier in the present study [51]. We tested the inhibitory effects of different NPs, the specific procedures were illustrated in Fig. 8A. H22-bearing mice undergoing IRFA were divided randomly into five groups (PBS, STF, BZP, SZP, and SBZP) and administered with NPs (concentration of 4 mg/kg STF and 2 mg/kg BMS) via the tail vein every 2 days for 6 consecutive times. Western blot and immunofluorescence were used to verify the activation of autophagy induced by NPs. The results demonstrated that the level of LC3B in SZP and SBZP treatment groups obviously increased (Fig. 8B and Additional file 2: Fig. S15). Next, the curve of tumor volume and bioluminescent imaging (Fig. 8C, E, H) revealed that the BZP group exhibited a mild inhibitory effect on the residual tumor. This undesirable outcome indicated that the residual tumors after IRFA had poor sensitivity to anti-PD-1/PD-L1 therapy. In contrast, the SZP and SBZP groups displayed a significant inhibitory effect on residual tumors. Both SZP and SBZP initially performed well in inhibiting tumors. However, the residual tumors regrew slowly 22 days after the treatment of SZP. Therefore, using SZP alone was incapable of achieving lasting outcomes. Contrastively, the sustained suppression of residual tumors in the SBZP group indicated that STF could enhance the sensitivity of residual tumors to anti-PD-1/PD-L1 therapy. On day 24, all mice were euthanized, and tumors were extracted and weighed (Fig. 8D). The results showed that in comparison with the control group, the tumor inhibition rates of the BZP, SZP, and SBZP groups were 29.62%, 71.00%, and 86.76%, respectively (Fig. 8F). In addition, we evaluated the influence of different NPs on tumor proliferation and apoptosis. It turned out that the proportion of Ki-67^+^ decreased and TUNEL^+^ increased in the SBZP group (Fig. 8G), illustrating that SBZP was able to considerably inhibit tumor proliferation and contribute to tumor apoptosis. These data suggest that SBZP NPs can exert a strong inhibitory effect on the growth of residual tumors and combination therapy has a more significant outcome.

There was no significant change in the body weight of the mice in each group (Fig. 8I), which indicated that NPs (SZP, BZP, and SBZP) induced few side effects. H&E staining of major organs revealed no obvious histopathological changes (Additional file 2: Fig. S17). Biochemical blood examination (Additional file 1: Fig. S9) and blood routine (Additional file 1: Fig. S10) also indicated that SBZP NPs were biocompatible.

### SBZP remodels the immune microenvironment of residual tumors after IRFA

**Fig. 9:**
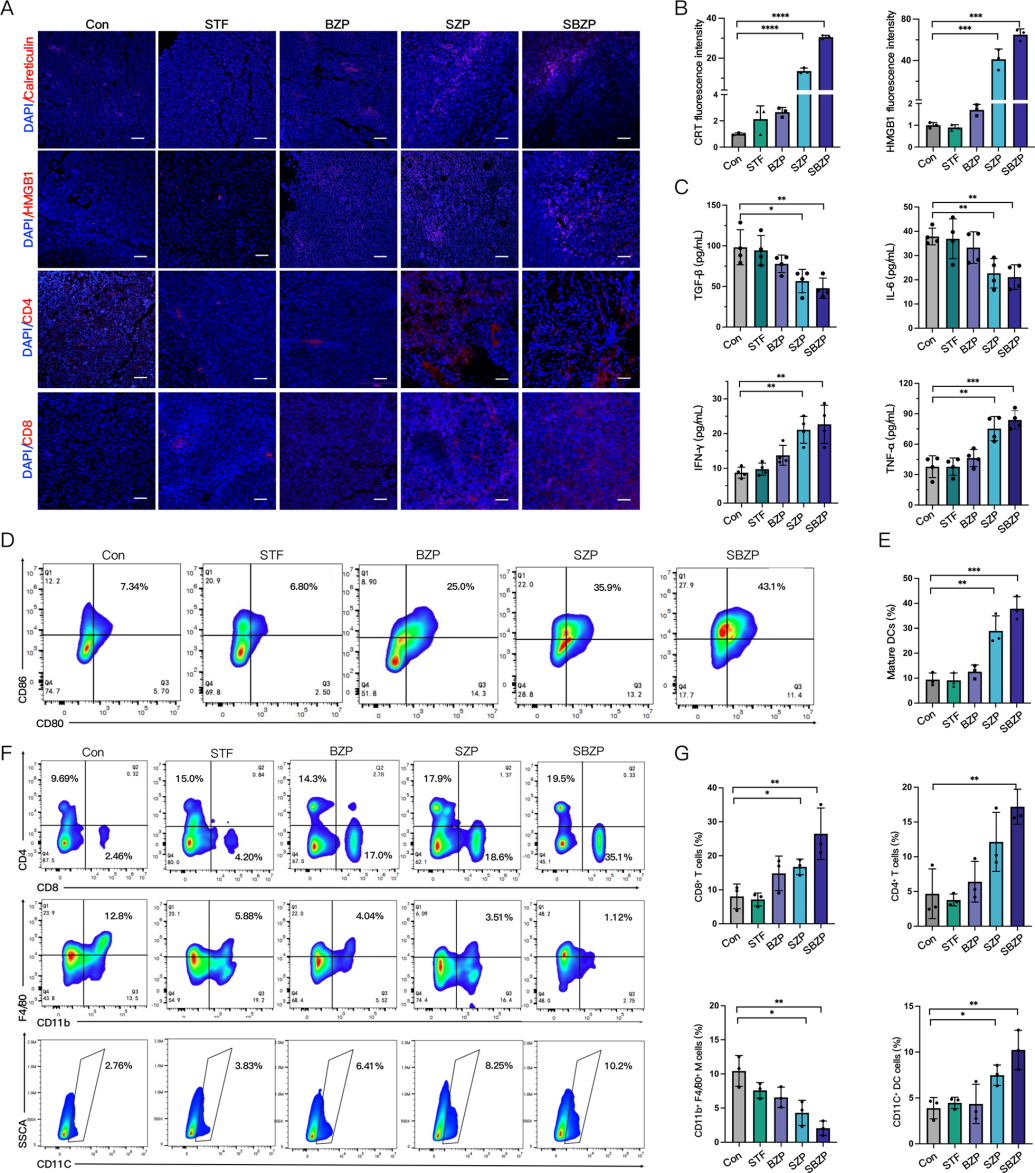
SBZP remodels the immune microenvironment of residual tumors after IRFA. **A**, **B** The expression and quantitative analysis of CRT and HMGB1 in residual tumors treated with different NPs via CLSM (n = 3). **C** Levels of IFN-γ, TNF-α, IL-6, and TGF-β in IRFA mice serum treated with different NPs by ELISA (n = 4). **D**, **E** The maturation and quantitative analysis of DCs in tumor-draining lymph nodes in IRFA mice treated with different NPs via flow cytometry (n = 3). **F**, **G** Representative flow cytometry plots and quantitative assessments of CD4^+^ T cells, CD8^+^ T cells, CD11C^+^ DCs and CD11b^+^/F4/80^+^ TAMs (of CD45^+^ gate) in residual tumor treated with different NPs (n = 3). Results were expressed as mean ± SD, *p < 0.05, **p < 0.01, ***p < 0.001, and ****p < 0.0001

The modulatory effect on the tumor immune microenvironment was examined to explore its potential antitumor mechanism of SBZP. We found that SBZP treatment boost CRT exposure and HMGB1 release in tumor tissues (Fig. 9A, B), and induced the maturation of DCs in the TDLN with a level 3.64 times and 5.10 times that of the control group (Fig. 9D, E). In addition, we discovered that SBZP activated systemic T cell anti-tumor immune responses. Immunofluorescence staining (Fig. 9A), immunohistochemistry (Additional file 2: Fig. S16), and flow cytometry (Fig. 9F) detected prominent enhancement in the tumor infiltration of CD4^+^ T cells, CD8^+^ T cells, and CD11C^+^ DCs cells in the SBZP group. According to flow cytometry, the proportion of TAMs infiltration after SBZP treatment was obviously reduced, lower by 71.5% and 81.1%, respectively (Fig. 9G) in comparison with the control group. Furthermore, an obvious increase in tumor-killing cytokine (IFN-γ, TNF-α) and a decrease in tumor-promoting cytokines (TGF-β, IL-6) in the serum of SBZP-treated mice were found by ELISA (Fig. 9C). In conclusion, SBZP NPs can activate ICD, reverse the immunosuppressive microenvironment, and activate anti-tumor immune response after IRFA. They can serve as promising immunotherapy for inhibiting residual tumors after IRFA.

## Conclusion

In summary, we confirmed that IRFA induced protective autophagy and aggravated the immunosuppressive microenvironment. To this end, we successfully constructed SBZP NPs, which prominently inhibited the progression of residual tumors after IRFA. Our study provided a new therapeutic modality that combining the amplification of autophagy with ICB. Through this strategy, SBZP could convert protective autophagy after IRFA to ACD by enhancing autophagy, triggering the release of immunogenicity and evoking ICD. Hence, SBZP shows great performance in remodeling the immune microenvironment and strengthening immune surveillance. It can be concluded that this new modality is endowed with great potential in treating residual tumors after IRFA.

### Supplementary Information

**Additional file 1: S1.**. The microstructure of sublethally heated cells displayed in ordinary microscope. Scale: 100 µm. **S2, S5.** Representative IHC images of LC3B, CD4 and CD8 in tumors before, 3 days, and 8 days after IRFA. Scale: 100 µm. **S3, S4.**. Representative flow cytometry plots and quantitative analysis of CD4^+^ T cells, CD8^+^ T cells, CD11C^+^ DCs and CD11b^+^/F4/80^+^ TAMs (of the CD45^+^ gate) in the spleen of mice before, 3 days, and 8 days after IRFA (n = 3). **S6.** The dose-response curve for CCK8 assay of IC50 of STF62247 in different concentrations treating cells at 37 °C and 47 °C for 48 h (n = 3). **S7.** The dose-response curve for CCK8 assay of IC50 of STF62247 and SZP NPs in different concentrations treating cells at 47 °C for 48 h (n = 3). **S8.** Quantitative analysis of autophagosomes (yellow dots) and autolysosomes (red dots) in sublethally heated SMMC7721 and Huh7 cells observed by CLSM (n = 8). **S9, S10.** Comparison of the blood biochemical (ALT, ALP, CREA, UA, TP, ALB) (n = 9) and blood routine (RBC, WBC, HGB) (n = 6) between the control group and the SBZP group. **S11, S12.** Clone formation and quantitative analysis of sublethally heated SMMC7721 and Huh7 cells under the influence of different groups (n = 3).

**Additional file 2: S13.** Flow cytometry analysis of CRT expression in sublethally heated HCC cells and ATG5-knockdown HCC cells treated with SZP. **S14.** Quantitative analysis of CRT in sublethally heated HCC cells and ATG5-knockdown HCC cells treated with SZP by CLSM (n = 3). **S15, 16.** Representative IHC images of LC3B, P62, CD4, and CD8 expression in tumor treated with different NPs. **S17.** H&E staining of the mice’s main organs treated with different NPs. Results were expressed as mean ± SD, *p < 0.05, **p < 0.01, and ***p < 0.001.

## Data Availability

Data will be made available on request.

## Abbreviations

HCC: Hepatocellular carcinoma
IRFA: Incomplete radiofrequency ablation
ICB: Immune checkpoint blockade
TAMs: Tumor-associated macrophages
DAMP: Damage-associated molecular patterns
TME: Tumor microenvironment
ACD: Autophagy-dependent cell death
ICD: Immunogenic cell death
EPR: Augmented permeability and retention
STF: STF62247
BMS: BMS202
BMDCs: Bone marrow-derived dendritic cells
3-MA: 3-Methyladenine
CQ: Chloroquine
IC50: 24H half maximal inhibitory concentration
CRT: Calreticulin
TDLN: Tumor-draining lymph nodes
TEM: Effector memory T cell
